# Personal

**Published:** 1893-01

**Authors:** 


### Personal.

DR. W. S. RENNER, of Buffalo, sailed for Europe by the Red Star Line, on Wednesday, December 21, 1892. He was accompanied by Mrs. Renner, and will remain abroad for three months, during which time he will attend the important clinics in Great Britain and on the Continent.

DR. G. E. DE SCHWEINITZ has been elected Clinical Professor of Ophthalmology in the Jefferson Medical College, Philadelphia. This election is a substantial promotion for Dr. de Schweinitz, who was Professor of Ophthalmology in the Polyclinic, and Lecturer on Medical Ophthalmoscopy in the University of Pennsylvania.

Dr. Richard Douglas, of Nashville, Tenn., was elected President of the Tri-States (Ala., Ga., and Tenn.,) Medical Society, at its meeting held in Chattanooga, in November, 1892.

